# Refractory ocular mucous membrane pemphigoid treated successfully with upadacitinib

**DOI:** 10.1111/ddg.15848

**Published:** 2025-08-27

**Authors:** Vincenzo Maione, Eliana Forbice, Luca Bettolini, Carola Romanò, Stefano Bighetti, Sara Rovaris, Vito Romano

**Affiliations:** ^1^ Dermatology Department University of Brescia ASST Spedali Civili di Brescia Brescia Italy; ^2^ Department of Medical and Surgical Specialties Radiological Sciences and Public Health Ophthalmology Clinic University of Brescia Brescia Italy

Dear Editors,

Mucous membrane pemphigoid (MMP) is defined as pemphigoid disease with predominant mucosal involvement, with the ocular variant being the most severe due to scarring and visual impairment. While standard treatments can slow disease progression, options for treatment‐resistant cases are limited.^1^, ^2^ This report presents a case of mucous membrane pemphigoid successfully treated with upadacitinib.

A 72‐year‐old male was referred in February 2023 for the management of MMP. The diagnosis was confirmed through a biopsy of a cutaneous bullous lesion.

At presentation, the patient's visual acuity was 5/10 in the right eye (OD) and 2–3/10 in the left eye (OS). He exhibited marked thickening of the eyelid margins, severe ocular redness, extensive symblepharon, predominantly in the temporal quadrant, as well as corneal involvement with filamentary keratitis and cornea guttata (Figure 1a). His ocular condition was classified as Foster stage 3 and Mondino stage 3, indicating advanced cicatricial pemphigoid.

**FIGURE 1:**
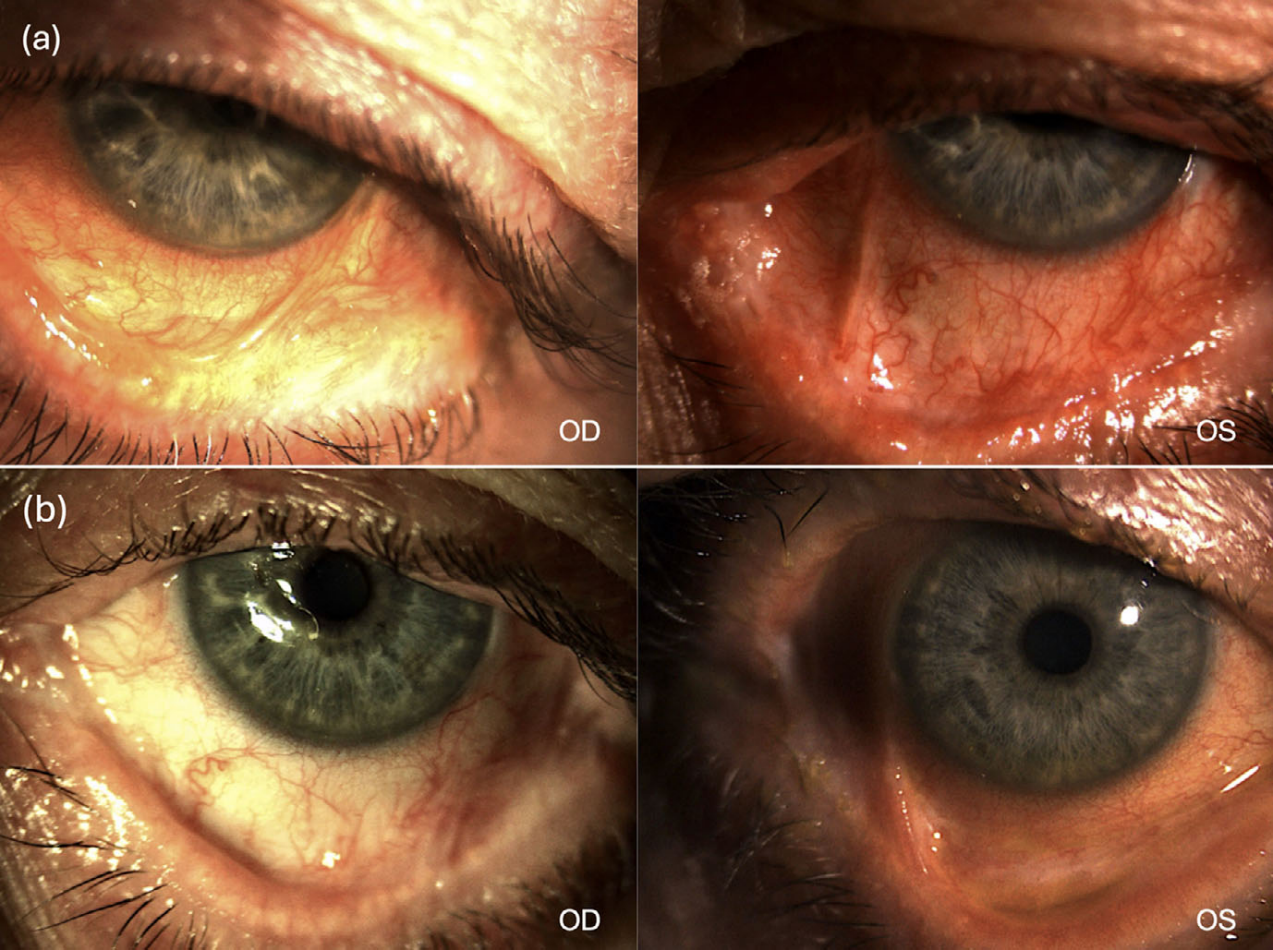
(a) Initial presentation showing severe blepharitis, trichiasis due to subepithelial scarring, and keratinization of the eyelid margin. Symblepharon formation is evident with flattening of the plica and keratinization of the caruncle (Stage III of Foster's Classification). Corneal involvement is significant, with diffuse punctate epitheliopathy and signs of chronic inflammation. (b) Post‐treatment improvement following upadacitinib therapy. The eyelid margin appears less inflamed, with reduction of blepharitis and distichiasis. Ocular redness is markedly decreased, and corneal punctate epitheliopathy has improved, indicating a positive response to therapy. *Abbr*.: OD, oculus dexter; OS, oculus sinister

The patient was initially treated with dapsone (100 mg/day) and subsequently prednisone (60 mg/day) was added. Due to incomplete response, dapsone was replaced with mycophenolate mofetil (2 g/day), but inflammation persisted despite this regimen. In an attempt to achieve better disease control, rituximab (two 1,000 mg infusions, 2 weeks apart) was administered, leading to minimal clinical improvement. Subsequently, intravenous immunoglobulin (IVIG) cycles (2 g/kg per cycle) were associated, but despite this therapy, corneal involvement remained significant.

In July 2023, after an inadequate response to therapy, discontinuation of all previous immunosuppressive agents (mycophenolate mofetil, intravenous immunoglobulins, and oral corticosteroids), and the patient's refusal of alternatives (cyclophosphamide because of side effects and etanercept because of needle aversion), treatment was switched to upadacitinib 15 mg/day as monotherapy, which was later escalated to 30 mg/day because of a partial response. By the fourth week of treatment, the patient experienced a significant reduction in ocular redness and inflammation, along with marked symptomatic relief from burning and discomfort (Figure 1b). At follow‐up, visual acuity had improved to 6/10 in the OD and 7/10 in the OS, further increasing to 10/10 with pinhole correction. However, potential visual acuity remained limited due to concurrent lens sclerosis and retinal pigment epithelial changes. No adverse effects have been reported so far.

Upadacitinib is an orally administered Janus kinase (JAK)1 inhibitor approved for use in various immune‐mediated inflammatory diseases.^3^ In MMP, pro‐inflammatory cytokines, including interleukin (IL)‐1, tumor necrosis factor (TNF)‐alpha, IL‐6 and IL‐13 are found to be elevated in the conjunctival tissues of patients.^4^, ^5^ JAK1 inhibition blocks TNF‐alpha production and suppresses Th2 immunity, both crucial in the pathogenesis of the disease.^6^ Notably, its inhibitory effect on IL‐13, a cytokine implicated in progressive conjunctival fibrosis, may help control fibrosis even during clinical quiescence.

Previous case reports have documented the use of JAK inhibitors in MMP, primarily focusing on tofacitinib and baricitinib in combination with other immunosuppressive agents, such as methotrexate.^6^ Only one case report describes the use of abrocitinib.^7^ Tofacitinib, a pan‐JAK inhibitor, has demonstrated efficacy in controlling inflammation and halting disease progression in refractory cases of ocular MMP, though its safety profile requires careful monitoring, particularly due to an increased risk of infections and thrombosis.^8^ Baricitinib, a JAK1/2 inhibitor, has also shown promising results when combined with methotrexate in recalcitrant multilocular MMP, with reports indicating significant improvement in mucosal involvement and systemic manifestations.^6^

Compared to these agents, upadacitinib, a selective JAK1 inhibitor, offers advantages including a more targeted mechanism of action, a potentially better safety profile than tofacitinib, and elimination of the need for methotrexate co‐administration, as seen with baricitinib.^6^ However, it is important to note that this disease primarily affects elderly patients, often with comorbidities. These factors must be considered when prescribing JAK‐STAT inhibitors, which carry a risk particularly for patients with cardiovascular diseases or a predisposition to thromboembolic events or herpes virus infections.^9^

In conclusion, we report the use of upadacitinib in a case of MMP with ocular involvement, suggesting that JAK inhibition may be a promising therapeutic option for patients, particularly when conventional immunosuppression fails. Further studies are necessary to assess its long‐term efficacy and potential role in preventing complications such as corneal scarring and symblepharon formation.

## CONFLICT OF INTEREST STATEMENT

None.
